# Rising incidence of severe maxillofacial space infections in Germany

**DOI:** 10.1007/s00784-024-05663-w

**Published:** 2024-04-22

**Authors:** Axel Meisgeier, Simon Pienkohs, Florian Dürrschnabel, Andreas Neff, Frank Halling

**Affiliations:** 1 Department of Oral and Craniomaxillofacial Surgery, UKGM GmbH, University Hospital Marburg and Faculty of Medicine, Philipps University, 35043 Marburg, Germany; 2 Gesundheitszentrum Fulda, Praxis für MKG-Chirurgie, Gerloser Weg 23a, D-36039 Fulda, Germany

**Keywords:** Epidemiology, Incision, Maxillofacial abscess, Odontogenic abscess, Super octogenarian, Time trends

## Abstract

**Objectives:**

Severe maxillofacial space infection (MSI) as an end stage of dentoalveolar diseases or complication of sialadenitis is a potentially life-threatening disease accompanied by complications including airway obstruction, jugular vein thrombosis, descending mediastinitis, sepsis and acute respiratory distress syndrome. The aim of this study was to analyze the incidence and time trends of severe MSI and potentially influencing factors in the German healthcare system over time.

**Materials and methods:**

Nationwide data regarding the national diagnosis-related-group (DRG) inpatient billing system was received from the German Federal Statistical Office. A retrospective analysis of incidence and time trends of MSI-associated procedures classified with the Operation and Procedure Classification System (OPS), were statistically evaluated using Poisson regression analysis between 2005 and 2022 and were associated with different epidemiological factors.

**Results:**

The total standardized incidence rate of MSI-associated procedures in the observational period 2005—2022 was 9.8 (♀8.2; ♂11.4) per 100,000 person years. For all age groups a significant increase of 46.1% in severe MSI – related surgical interventions was registered within the observational period. The largest increase (120.5%) was found in elderly patients over 80 years. There were significant differences of the incidences of MSI-associated surgeries between the different federal states in Germany.

**Conclusions:**

Severe MSI are a growing challenge in German health care especially among elderly patients over 80 years.

**Clinical relevance:**

Severe MSI is a promising target for prevention. There should be more focus in primary dental and medical care especially in groups depending on social support.

## Introduction

Severe maxillofacial space infections (MSI) are defined as cellulitis or abscess formations of the fascial planes and potential spaces of the head and neck [1]. Early identification and therapy are crucial in prevention of severe potential life-threatening complications such as upper airway obstruction, descending mediastinitis, pleural empyema, pericarditis, jugular vein thrombosis, sepsis or carotid pseudoaneurysm [2]. Even lethal outcomes were documented [3, 4]. Most common primary sources of MSI are end stage of dental disease, complication of sialadenitis, perioperative complication or malignancy [5]. Microbiology typically reveals a mixed bacterial flora, including anaerobic species, that can rapidly progress. Usually, the results of cultures are polymicrobial, and are often caused by oral bacteria. Streptococci are the organisms most commonly cultured from MSI [6]. Since the main symptoms of infection are similar between the different etiologies, clinical diagnosis may be difficult [7]. Identification of causative factors may require radiological examination with ultrasound (US), computed tomography (CT) or magnetic resonance imaging (MRI). Historically, with the introduction and widespread availability of antibiotics the incidence and severity of this disease have declined dramatically. The wide availability of dental care is also an important factor in prevention of MSI [8, 9].

The pillars of treatment are appropriate antimicrobial therapy, sufficient surgical drainage, endodontic or surgical treatment of causative teeth as well as the early management of complications [5, 10]. A calculated empirical antibiotic treatment is necessary until culture-guided antimicrobial therapy is available [11]. Improving the immunological status can have a significantly positive influence on the course of the disease in patients with systemic risk factors such as diabetes mellitus, end stage renal disease or autoimmune disorders [12]. Especially in an aging society with increasing multimorbidity and social imbalance MSI continue to represent a challenge with potentially preventable mortality [13, 14]. However, population-based data about trends in the incidence of severe MSI due to its different causes are limited and may be inconclusive.

Studies from the UK report that the incidence of MSI has increased within the last decades as growing health inequalities are leading to an increase in hospital admissions due to severe odontogenic infections [15, 16]. To our knowledge, there is no conclusive data from any other central European country. The purpose of this study was therefore to investigate the nationwide incidence of severe MSI in Germany. For this purpose, the regional, gender-specific and age-related distribution of patients suffering from severe MSI who required inpatient external incision and drainage in different maxillofacial regions was evaluated.

## Materials and methods

The national diagnosis-related groups (DRG) inpatient billing system includes data from all hospitals in Germany that use the DRG system. More than 99% of inpatient treatments are covered. Hospitals are required by law to provide comprehensive information about hospital care, including patient demographics, diagnoses, comorbidities, complications and procedures.

Surgical procedures from the years 2005 to 2022 were coded according to the OPS (Operation and Procedure Classification System), a German modification of the ICPM (International Classification of Medical Procedures). All diagnoses were coded according to the ICD-10GM (German version of the International Classification of Diseases and Related Health Problems, 10th Edition). Detailed lists of all procedures regarding extraoral incision and drainage of severe MSI per year (coded 5–270.0 to 5–270.9; 5–270.x and 5–270.y in the OPS) were provided by the German Federal Statistical Office (Statistisches Bundesamt—Destatis. Genesis-Online. Data license by-2–0). MSI—associated procedures per year (PPY) were calculated and reported. Mean age of patients was calculated. The normality of the distribution of continuous variables was tested by Kolmogorov–Smirnov test. Continuous variables with normal distribution were presented as mean and standard deviation. Means of 2 continuous normally distributed variables were compared by independent samples Student's t-test. The differences were considered significant at *p* < 0.05. In addition, population-adjusted rates of MSI-associated surgeries per 100,000 person years were calculated using population data also provided by the German Federal Statistical Office and reported with 95% confidence interval. In order to avoid seasonal influences, the MSI-associated infection procedures were analyzed on an annual basis. We performed the main analysis for the entire population and the analyses stratified by sex, age group and federal state. We computed crude and age-sex standardized incidence rates of severe MSI for each calendar year, taking the German population of the latest census 2011 as standard population using 0–14, 15–34, 35–59, 60–79 and ≥ 80 years as age classes to exclude bias due to demographic heterogeneity over time. To ascertain male and female incidence rates, age standardization was performed with the aforementioned age classes using the age distribution of the male and female standard population resulting in different weights for both genders. The calculation of the 95% confidence intervals (95% CI) of crude and standardized incidences was performed by the delta method. Separate Poisson regression models were computed to investigate time trends, fitted with incidence rates of MSI as dependent variables and age group, gender, federal state and year of MSI starting from baseline year 2005 as independent variables. The age class 15–34 years, female sex and federal state of Baden-Württemberg were used as reference group. All models were adapted by descale adjustment to account for overdispersion of the outcome variable. Statistical analysis was performed using IBM SPSS Statistics Version 29.0 (IBM Deutschland GmbH, Böblingen, Germany).

## Results

**Fig. 1:**
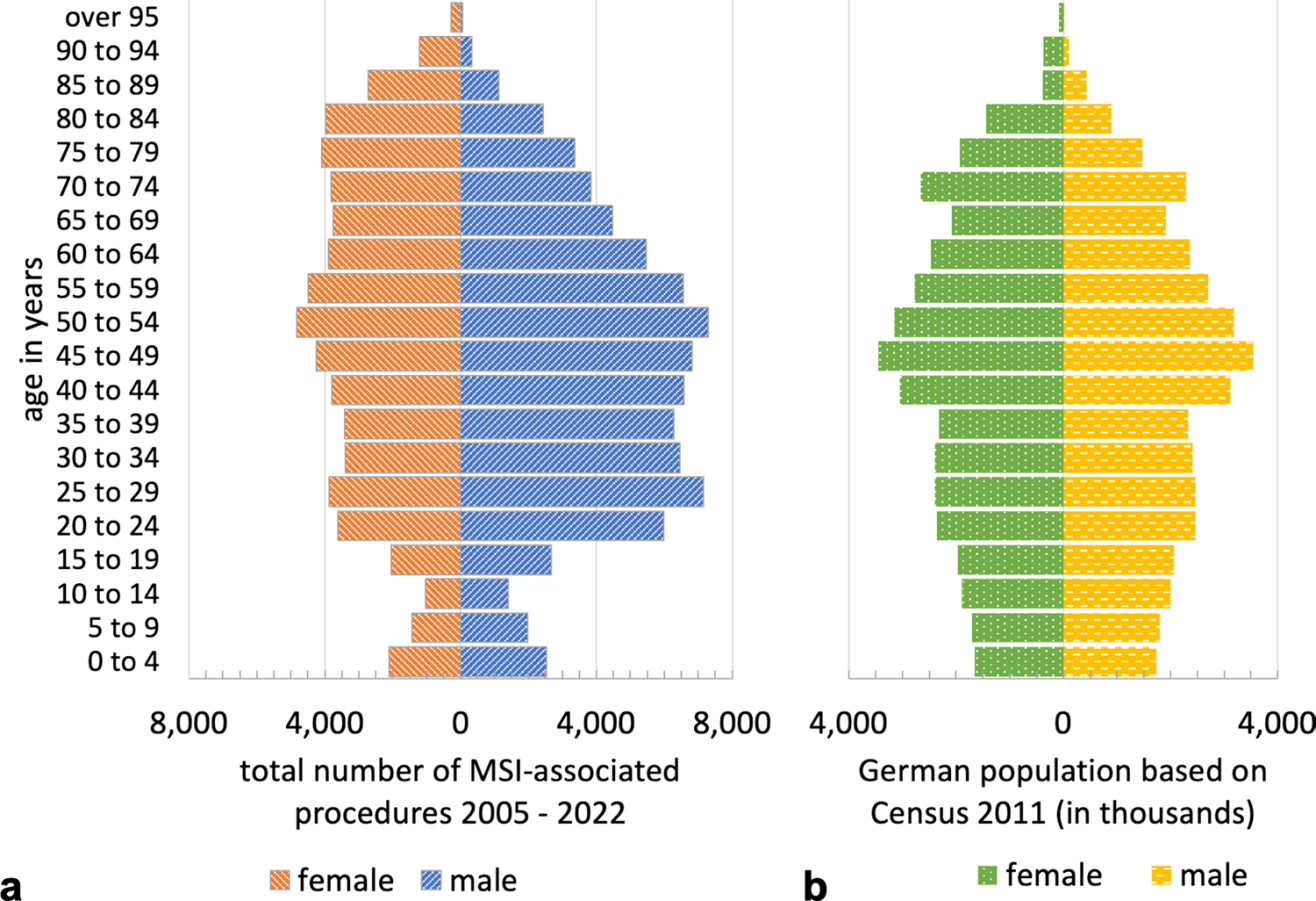
**a** Age-gender-diagram of the total number of MSI-associated procedures 2005–2022. **b** Age-gender-diagram of the German population based on census 2011

**Fig. 2:**
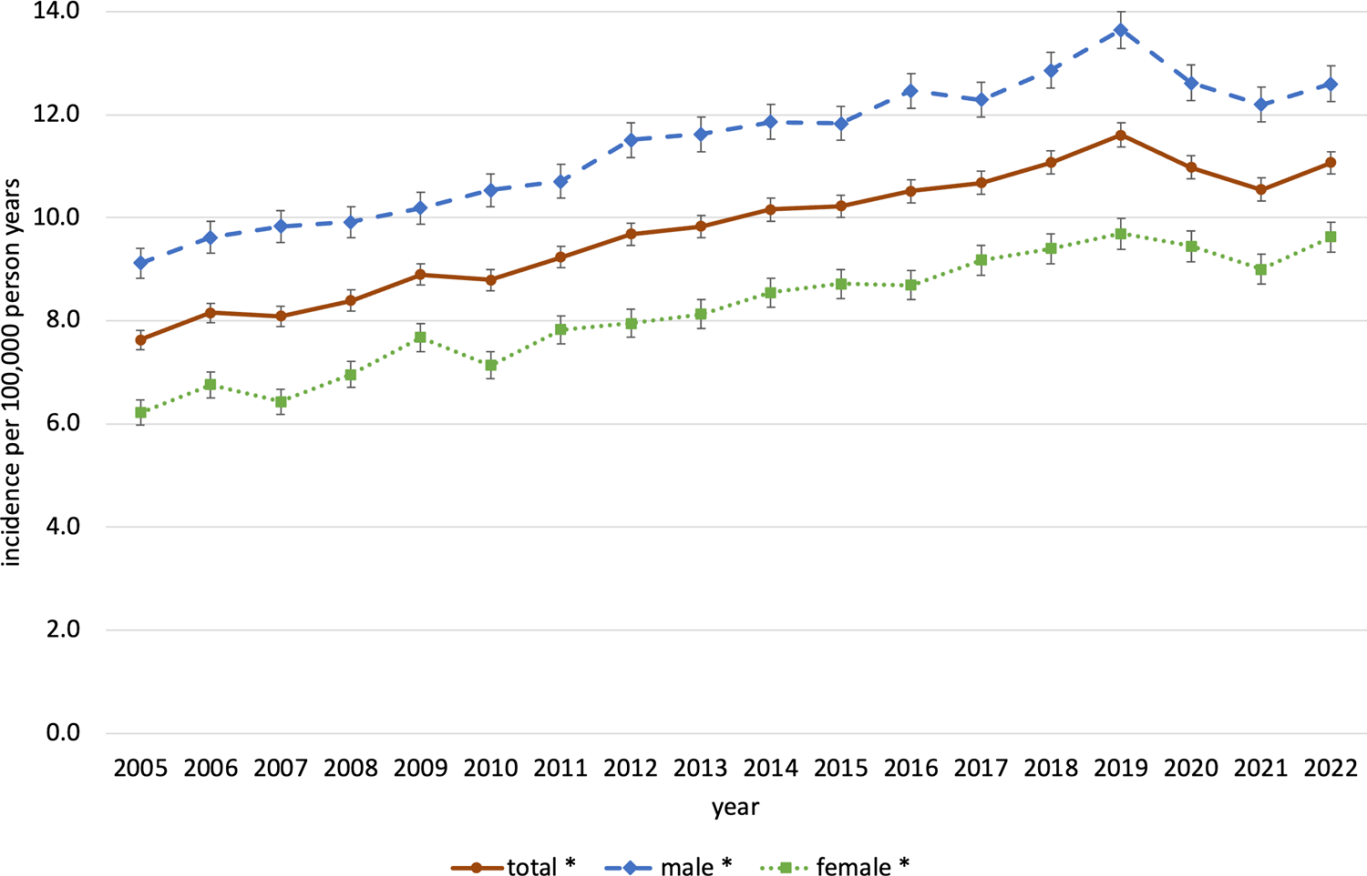
Standardized overall and gender-specific incidence of MSI-associated procedures, Germany, 2005–2022. *Significant increase (*p*-value time trend Poisson-Regression model < 0.05)

**Fig. 3:**
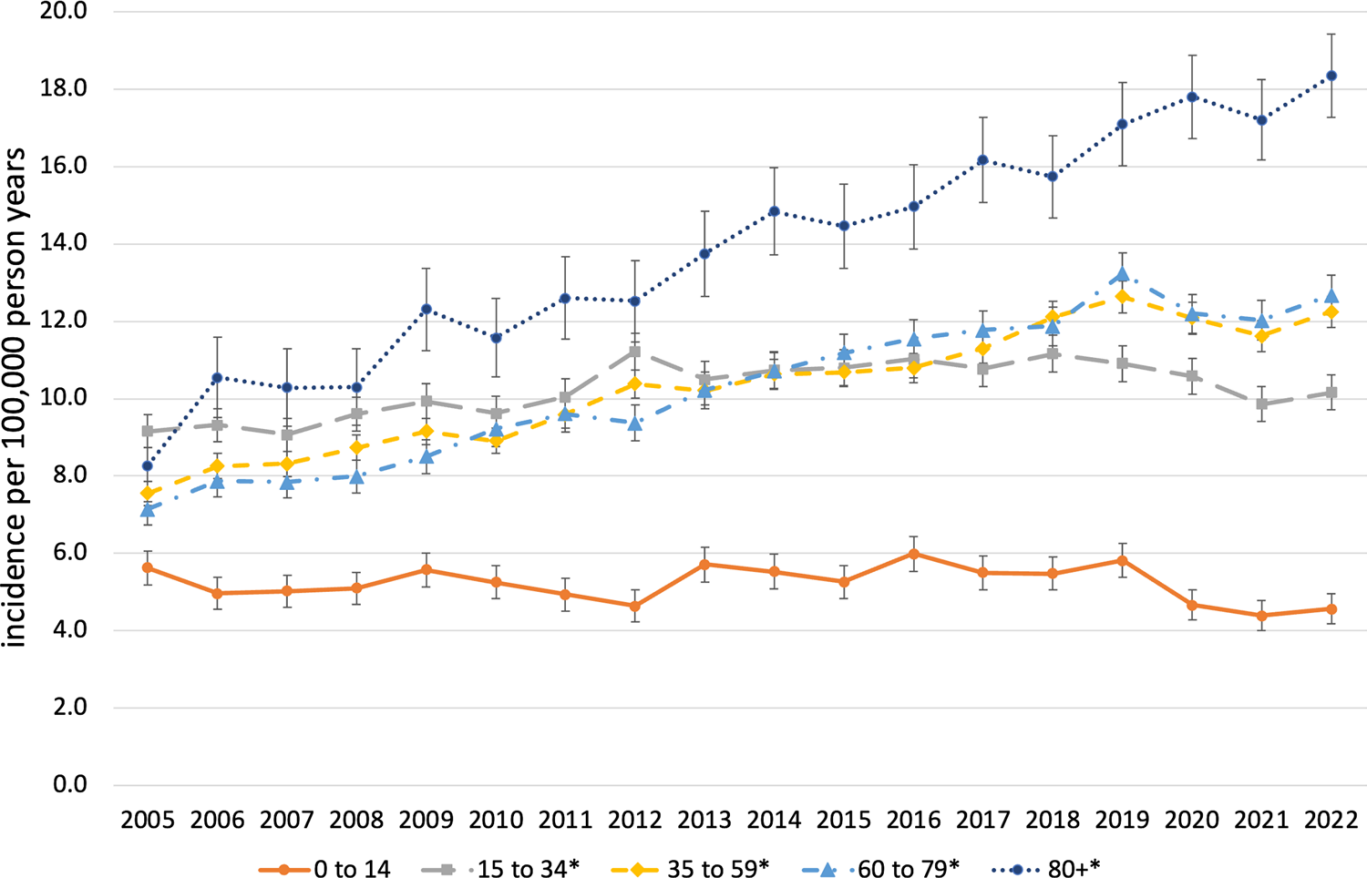
Standardized age-specific incidence of MSI-associated procedures, Germany, 2005–2022. *Significant increase (*p* value time trend Poisson-Regression model < 0.05)

In the observational period 2005—2022 a total of 144,881 MSI-associated procedures were registered in Germany and could be included in the study. The distribution regarding age, gender and year of cases treated for severe MSI is presented in Table 1 and Fig. 1. The majority of cases were older than 35 years (68.4%) and male (57.2%). The mean age increased within this period from 45.0 years between 2005 and 2013 to 48.6 years between 2014 and 2022 (*p* < 0.01). The age and gender standardized incidence rates of procedures associated with severe MSI are shown in Table 2, Figs. 2 and 3 for the total population and stratified by gender and age. The total standardized incidence rate of MSI-associated procedures in the observational period 2005—2022 was 9.8 per 100,000 person years [95% CI: 9.7–9.8] ranging from 7.6 [7.4–7.8] in 2005 to 11.6 [11.4–11.8] in 2019. In 2022 the incidence was 11.1 [10.9–11.3], an increase of + 45.7% within the observational period. Gender distribution slightly shifted from 59.5% in males and 40.5% in females in 2005 to 56.7% and 43.3% in 2022, respectively. In males the overall incidence was 11.4 [11.3–11.5] spreading from 9.1 [8.8–9.4] in 2005 to 13.6 [13.3–14.0] in 2019, in females the total incidence rate was 8.2 [8.1–8.3] varying between 6.2 [6.0–6.5] in 2005 and 9.7 [9.4–10.0] in 2019.

**Table 1:** Total numbers and mean age of MSI-associated procedures in Germany 2005 – 2022

All MSI-associated procedures		Total	Male	Female
Number of procedures mean age (years, SD)		144,881 47.2 (22.2)	82,825 44.7 (20.7)	62,053 50.5 (23.8)
Year				
2005	number of procedures mean age (years, SD)	6282 42.1 (21.9)	3687 40.2 (20.0)	2594 44.7 (24.2)
2006	number of procedures mean age (years, SD)	6669 43.8 (21.9)	3866 41.6 (19.9)	2802 46.9 (24.1)
2007	number of procedures mean age (years, SD)	6630 44.2 (21.9)	3957 42.2 (20.2)	2673 47.2 (23.9)
2008	number of procedures mean age (years, SD)	6884 44.3 (21.7)	3993 42.2 (20.0)	2891 47.3 (23.5)
2009	number of procedures mean age (years, SD)	7282 44.9 (22.2)	4094 42.3 (20.5)	3188 48.4 (23.9)
2010	number of procedures mean age (years, SD)	7199 45.8 (22.2)	4235 43.3 (20.5)	2964 49.4 (24.1)
2011	number of procedures mean age (years, SD)	7420 46.7 (22.1)	4202 44.0 (20.4)	3218 50.2 (23.6)
2012	number of procedures mean age (years, SD)	7814 46.1 (21.6)	4539 44.0 (20.0)	3275 49.0 (23.3)
2013	number of procedures mean age (years, SD)	7970 46.7 (22.3)	4611 44.5 (20.6)	3358 49.8 (24.1)
2014	number of procedures mean age (years, SD)	8300 47.5 (22.2)	4746 45.1 (20.8)	3554 50.8 (23.7)
2015	number of procedures mean age (years, SD)	8462 47.6 (22.3)	4803 44.7 (20.7)	3659 51.3 (23.7)
2016	number of procedures mean age (years, SD)	8739 47.5 (22.6)	5070 44.7 (21.2)	3669 51.3 (23.9)
2017	number of procedures mean age (years, SD)	8905 48.4 (22.4)	5012 45.8 (21.0)	3893 51.9 (23.7)
2018	number of procedures mean age (years, SD)	9228 48.2 (22.4)	5234 45.8 (21.1)	3994 51.5 (23.7)
2019	number of procedures mean age (years, SD)	9710 49.3 (22.5)	5564 47.1 (21.3)	4146 52.1 (23.7)
2020	number of procedures mean age (years, SD)	9197 49.9 (22.4)	5131 47.4 (21.0)	4066 53.1 (23.7)
2021	number of procedures mean age (years, SD)	8828 50.4 (22.4)	4924 47.8 (20.7)	3904 53.7 (23.9)
2022	number of procedures mean age (years, SD)	9362 50.6 (22.3)	5157 48.2 (21.1)	4205 53.6 (23.4)
Age group				
0—14 years		10,498	5914	4584
15—34 years		35,222	22,276	12,946
35—59 years		54,304	33,525	20,779
60—79 years		32,696	17,153	15,543
Over 80 years		12,158	3957	8201

**Table 2:** Incidence of severe MSI-associated procedures in Germany 2005—2022. Incidence rates (95% confidence interval) per 100,000 person years standardized to the German population based on the Census 2011

	Total	Gender		Age group				
		Male	Female	0—14 years	15 – 34 years	35 – 59 years	60—79 years	over 80 years
All years	9.8 (9.7 – 9.8)	11.4 (11.3 – 11.5)	8.2 (8.1 – 8.3)	5.2 (5.1—5.3)	10.2 (10.1—10.4)	10.3 (10.2—10.4)	10.3 (10.2—10.4)	13.8 (13.6—14.1)
Year								
2005	7.6 (7.4 – 7.8)	9.1 (8.8—9.4)	6.2 (6.0—6.5)	5.6 (5.2—6.0)	9.2 (8.7—9.6)	7.5 (7.2—7.9)	7.1 (6.7—7.5)	8.3 (7.3—9.2)
2006	8.2 (8.0 – 8.3)	9.6 (9.3—9.9)	6.8 (6.5 – 7.0)	5.0 (4.6—5.3)	9.3 (8.9—9.7)	8.3 (7.9—8.6)	7.9 (7.4—8.3)	10.6 (9.5—11.6)
2007	8.1 (7.9 – 8.3)	9.8 (9.5—10.1)	6.4 (6.2—6.7)	5.0 (4.6—5.3)	9.1 (8.6—9.5)	8.3 (8.0—8.6)	7.8 (7.4—8.3)	10.3 (9.3—11.3)
2008	8.4 (8.2 – 8.6)	9.9 (9.6—10.2)	7.0 (6.7—7.2)	5.1 (4.7—5.4)	9.6 (9.2—10.0)	8.7 (8.4—9.1)	8.0 (7.6—8.4)	10.3 (9.3—11.3)
2009	8.9 (8.7 – 9.1)	10.2 (9.9—10.5)	7.7 (7.4—7.9)	5.6 (5.1—5.9)	9.9 (9.5—10.4)	9.2 (8.8—9.5)	8.5 (8.1—8.9)	12.3 (11.2—13.4)
2010	8.8 (8.6 – 9.0)	10.5 (10.2—10.9)	7.1 (6.9—7.4)	5.3 (4.8—5.6)	9.6 (9.2—10.1)	8.9 (8.6—9.2)	9.2 (8.8—9.7)	11.6 (10.6—12.6)
2011	9.2 (9.0 – 9.4)	10.7 (10.4 – 11.0)	7.8 (7.6—8.1)	4.9 (4.5—5.3)	10.1 (9.6—10.5)	9.6 (9.2—9.9)	9.6 (9.1—10.1)	12.6 (11.5—13.7)
2012	9.7 (9.5 – 9.9)	11.5 (11.2—11.8)	7.9 (7.7—8.2)	4.6 (4.2—5.0)	11.2 (10.7—11.7)	10.4 (10.0—10.7)	9.4 (8.9—9.8)	12.5 (11.5—13.6)
2013	9.8 (9.6 – 10.0)	11.6 (11.3 – 12.0)	8.1 (7.9—8.4)	5.7 (5.3—6.1)	10.5 (10.0—11.0)	10.2 (9.8—10.6)	10.2 (9.7—10.7)	13.8 (12.7—14.9)
2014	10.2 (9.9 – 10.4)	11.9 (11.5—12.2)	8.5 (8.3—8.8)	5.5 (5.1—5.9)	10.7 (10.3—11.2)	10.6 (10.3—11.0)	10.7 (10.2—11.2)	14.8 (13.7—16.0)
2015	10.2 (10.0 – 10.4)	11.8 (11.5—12.2)	8.7 (8.4 – 9.0)	5.3 (4.8—5.6)	10.8 (10.3—11.3)	10.7 (10.3—11.1)	11.2 (10.7—11.7)	14.5 (13.4—15.5)
2016	10.5 (10.3 – 10.7)	12.5 (12.1—12.8)	8.7 (8.4 – 9.0)	6.0 (5.5—6.3)	11.0 (10.6—11.5)	10.8 (10.4—11.2)	11.5 (11.0—12.0)	15.0 (13.9—16.0)
2017	10.7 (10.5 – 10.9)	12.3 (12.0—12.6)	9.2 (8.9—9.5)	5.5 (5.1—5.8)	10.8 (10.3—11.2)	11.3 (10.9—11.7)	11.8 (11.3—12.3)	16.2 (15.1—17.3)
2018	11.1 (10.8 – 11.3)	12.9 (12.5—13.2)	9.4 (9.1—9.7)	5.5 (5.0—5.8)	11.2 (10.7—11.6)	12.1 (11.7—12.5)	11.9 (11.4—12.4)	15.7 (14.7—16.8)
2019	11.6 (11.4 – 11.8)	13.6 (13.3 – 14.0)	9.7 (9.4—10.0)	5.8 (5.4—6.2)	10.9 (10.4—11.4)	12.6 (12.2—13.0)	13.2 (12.7—13.8)	17.1 (16.0—18.2)
2020	11.0 (10.8 – 11.2)	12.6 (12.3 – 13.0)	9.4 (9.2—9.7)	4.7 (4.3—5.0)	10.6 (10.1—11.1)	12.1 (11.7—12.5)	12.2 (11.7—12.7)	17.8 (16.7—18.9)
2021	10.5 (10.3 – 10.8)	12.2 (11.9—12.5)	9.0 (8.7—9.3)	4.4 (4.0—4.7)	9.9 (9.4—10.3)	11.6 (11.2—12.0)	12.0 (11.5—12.5)	17.2 (16.2—18.2)
2022	11.1 (10.8 – 11.3)	12.6 (12.3—12.9)	9.6 (9.3—9.9)	4.6 (4.2—4.9)	10.2 (9.7—10.6)	12.2 (11.8—12.7)	12.7 (12.2—13.2)	18.3 (17.3—19.4)

Among the different age groups children from 0 to 14 years had the lowest incidence rates with a total of 5.2 [5.1–5.3] ranging from 4.4 [4.0–4.7] in 2021 to 6.0 [5.5–6.3] in 2016. Among adolescents and young adults from 15 to 34 years the total incidence rate 10.2 [10.1–10.4] differing between 9.1 [8.6–9.5] in 2007 to 11.2 [10.7–11.7] in 2012. In middle aged adults from 35 to 59 years the overall incidence was 10.3 [10.2–10.4] spreading from 7.5 [7.2–7.9] in 2005 to 12.6 [12.2–13.0] in 2019. Older adults from 60 to 79 years showed an incidence rate of 10.2 (10.1–10.3) varying from 7.1 [6.7–7.5] in 2005 and 13.2 [12.7–13.8] in 2019. Super octogenarians (80 years and older) had the highest total incidence rate with 13.8 [13.6–14.1] ranging from 8.3 [7.3–9.2] in 2005 to 18.3 [17.3–19.4] in 2022 and the strongest increase of 120.5% during this period.

Looking at the 16 federal states individually the overall incidence of surgical procedures associated with severe MSI within the observational period ranged from 5.8 [5.6–6.0] in Schleswig–Holstein to 16.3 [15.8–16.9] in Saarland as shown in Table 3. A single year minimum was seen in Schleswig–Holstein in 2006 with 4.2 [3.4–4.9]. A single year maximum was registered in Saarland with 22.3 [19.4–25.2] in 2017.

**Table 3:** Regional incidence of severe MSI in all 16 federal states of Germany 2005—2022. Incidence rates (95% confidence interval) per 100,000 person years standardized to the German population based on the Census 2011

	Germany	Baden-Wuerttemberg	Bavaria	Berlin	Brandenburg	Bremen	Hamburg	Hesse	Mecklenburg-Vorpommern
All years	9.8 (9.7 – 9.8)	9.8 (9.6—9.9)	6.9 (6.8—7.0)	12.2 (11.9—12.5)	8.8 (8.5—9.1)	15.3 (14.6—16.0)	15.1 (14.7—15.6)	8.0 (7.9—8.2)	14.3 (13.9—14.7)
2005	7.6 (7.4 – 7.8)	7.1 (6.6—7.6)	5.9 (5.5—6.4)	10.9 (9.8—12.1)	7.5 (6.4—8.5)	12.7 (10.0—15.5)	9.9 (8.4—11.3)	4.6 (4.1—5.2)	8.3 (6.9—9.6)
2006	8.2 (8.0 – 8.3)	8.4 (7.9—9.0)	6.4 (6.0—6.9)	10.5 (9.4—11.6)	7.4 (6.3—8.4)	15.2 (12.2—18.1)	11.2 (9.6—12.7)	5.0 (4.4—5.6)	10.6 (9.1—12.2)
2007	8.1 (7.9 – 8.3)	8.5 (7.9—9.0)	5.9 (5.5—6.3)	10.0 (8.9—11.0)	7.6 (6.6—8.7)	11.9 (9.3—14.5)	10.0 (8.5—11.5)	6.2 (5.5—6.8)	9.2 (7.8—10.7)
2008	8.4 (8.2 – 8.6)	8.6 (8.1—9.2)	5.6 (5.2—6.0)	10.1 (9.0—11.1)	6.9 (5.9—7.9)	15.5 (12.5—18.5)	12.4 (10.7—14.0)	6.5 (5.9—7.2)	10.5 (9.0—12.1)
2009	8.9 (8.7 – 9.1)	9.3 (8.7—9.8)	5.8 (5.4—6.3)	11.1 (10.0—12.2)	6.9 (5.9—7.9)	16.1 (13.1—19.2)	15.0 (13.2—16.8)	6.8 (6.1—7.4)	12.2 (10.5—13.9)
2010	8.8 (8.6 – 9.0)	8.6 (8.0—9.1)	5.8 (5.4—6.2)	11.5 (10.4—12.6)	8.1 (7.0—9.2)	14.1 (11.3—17.0)	12.9 (11.3—14.6)	7.6 (6.9—8.3)	13.1 (11.3—14.8)
2011	9.2 (9.0 – 9.4)	9.3 (8.7—9.8)	6.4 (6.0—6.8)	11.7 (10.5—12.8)	7.8 (6.7—8.9)	12.1 (9.4—14.8)	14.8 (13.0—16.6)	8.1 (7.4—8.9)	12.8 (11.1—14.6)
2012	9.7 (9.5 – 9.9)	10.0 (9.4—10.6)	6.6 (6.1—7.0)	11.8 (10.6—12.9)	8.3 (7.2—9.5)	13.2 (10.4—16.0)	13.3 (11.6—15.0)	8.8 (8.1—9.6)	14.8 (12.9—16.7)
2013	9.8 (9.6 – 10.0)	9.9 (9.3—10.5)	6.8 (6.4—7.3)	12.6 (11.5—13.8)	9.7 (8.4—10.9)	14.7 (11.8—17.6)	16.0 (14.2—17.9)	8.2 (7.5—9.0)	17.0 (15.0—19.1)
2014	10.2 (9.9 – 10.4)	9.8 (9.2—10.4)	6.8 (6.4—7.3)	13.0 (11.8—14.2)	8.2 (7.1—9.4)	16.4 (13.3—19.5)	16.1 (14.2—18.0)	8.1 (7.4—8.8)	17.4 (15.4—19.5)
2015	10.2 (10.0 – 10.4)	10.4 (9.8—11.0)	7.5 (7.0—8.0)	13.2 (12.0—14.4)	9.2 (8.0—10.4)	17.0 (13.9—20.1)	16.5 (14.6—18.4)	8.4 (7.7—9.1)	14.4 (12.6—16.3)
2016	10.5 (10.3 – 10.7)	10.9 (10.3—11.5)	7.5 (7.0—8.0)	12.4 (11.2—13.5)	8.4 (7.2—9.5)	17.0 (13.9—20.1)	19.6 (17.5—21.6)	8.8 (8.1—9.6)	15.5 (13.6—17.4)
2017	10.7 (10.5 – 10.9)	11.3 (10.7—11.9)	7.3 (6.8—7.7)	12.5 (11.3—13.6)	10.6 (9.3—11.8)	15.2 (12.2—18.1)	17.2 (15.3—19.1)	9.3 (8.5—10.0)	14.7 (12.9—16.6)
2018	11.1 (10.8 – 11.3)	11.4 (10.8—12.0)	7.6 (7.2—8.1)	12.2 (11.1—13.4)	11.0 (9.7—12.3)	18.2 (15.0—21.4)	20.1 (18.0—22.1)	8.9 (8.2—9.7)	17.2 (15.1—19.2)
2019	11.6 (11.4 – 11.8)	11.4 (10.7—12.0)	8.9 (8.4—9.4)	14.3 (13.1—15.5)	10.8 (9.6—12.1)	15.7 (12.7—18.7)	17.9 (15.9—19.8)	9.9 (9.1—10.6)	16.9 (14.9—18.9)
2020	11.0 (10.8 – 11.2)	10.6 (10.0—11.2)	7.7 (7.3—8.2)	14.5 (13.3—15.7)	9.9 (8.6—11.1)	17.2 (14.1—20.3)	14.8 (13.1—16.6)	10.2 (9.4—11.0)	19.4 (17.3—21.6)
2021	10.5 (10.3 – 10.8)	10.2 (9.6—10.8)	7.4 (6.9—7.9)	12.4 (11.2—13.5)	9.9 (8.7—11.2)	17.5 (14.4—20.7)	16.8 (15.0—18.7)	9.1 (8.4—9.9)	16.0 (14.1—18.0)
2022	11.1 (10.8 – 11.3)	10.0 (9.5—10.6)	7.9 (7.4—8.4)	14.7 (13.5—16.0)	10.2 (9.0—11.4)	15.7 (12.7—18.6)	18.0 (16.1—19.9)	9.9 (9.2—10.7)	17.4 (15.3—19.4)

	Lower Saxony	North Rhine- Westphalia	Rhineland-Palatinate	Saarland	Saxony	Saxony-Anhalt	Schleswig-Holstein	Thuringia
All years	8.9 (8.7—9.0)	10.8 (10.7—10.9)	9.8 (9.5—10.0)	16.3 (15.8—16.9)	9.0 (8.8—9.3)	15.0 (14.6—15.4)	5.8 (5.6—6.0)	11.1 (10.8—11.5)
2005	7.6 (7.0—8.2)	9.1 (8.7—9.6)	8.1 (7.2—8.9)	11.0 (9.0—13.0)	7.0 (6.2—7.8)	8.9 (7.7—10.1)	4.2 (3.4—4.9)	5.3 (4.4—6.3)
2006	7.0 (6.4—7.6)	9.6 (9.2—10.1)	8.0 (7.1—8.8)	14.4 (12.1—16.7)	6.4 (5.6—7.2)	10.2 (8.9—11.4)	5.4 (4.6—6.3)	6.4 (5.4—7.4)
2007	6.8 (6.2—7.4)	9.4 (8.9—9.8)	8.8 (7.9—9.7)	13.1 (10.9—15.3)	6.0 (5.3—6.8)	10.9 (9.6—12.2)	4.8 (4.0—5.6)	9.0 (7.8—10.3)
2008	7.4 (6.8—8.0)	9.6 (9.1—10.0)	8.6 (7.7—9.5)	14.4 (12.0—16.7)	7.0 (6.2—7.8)	12.9 (11.4—14.3)	5.3 (4.4—6.1)	7.9 (6.7—9.0)
2009	8.2 (7.5—8.8)	10.1 (9.7—10.6)	9.5 (8.6—10.5)	14.1 (11.8—16.4)	5.9 (5.2—6.7)	14.1 (12.6—15.6)	5.1 (4.3—5.9)	8.7 (7.5—9.9)
2010	8.6 (8.0—9.3)	9.5 (9.0—9.9)	8.5 (7.6—9.4)	13.7 (11.4—15.9)	7.6 (6.7—8.4)	14.6 (13.0—16.1)	5.0 (4.2—5.8)	10.2 (8.8—11.5)
2011	8.8 (8.2—9.5)	10.1 (9.6—10.5)	8.2 (7.3—9.1)	12.0 (9.9—14.2)	8.5 (7.6—9.4)	15.5 (13.9—17.1)	4.4 (3.6—5.2)	10.6 (9.3—12.0)
2012	8.4 (7.8—9.0)	10.8 (10.3—11.3)	9.1 (8.2—10.1)	14.0 (11.7—16.3)	8.5 (7.6—9.4)	17.4 (15.6—19.1)	5.1 (4.2—5.9)	12.1 (10.6—13.6)
2013	8.5 (7.9—9.2)	10.6 (10.1—11.1)	10.7 (9.7—11.8)	15.0 (12.6—17.4)	8.9 (8.0—9.8)	13.1 (11.6—14.6)	5.3 (4.5—6.2)	12.2 (10.7—13.6)
2014	9.8 (9.1—10.5)	11.4 (10.9—11.9)	8.9 (8.0—9.9)	16.3 (13.8—18.8)	9.8 (8.8—10.8)	16.8 (15.1—18.5)	5.7 (4.8—6.6)	12.4 (10.9—13.9)
2015	8.7 (8.0—9.3)	10.8 (10.3—11.3)	9.7 (8.8—10.7)	19.0 (16.3—21.7)	10.5 (9.5—11.5)	16.2 (14.6—17.9)	6.7 (5.7—7.6)	13.2 (11.6—14.7)
2016	9.1 (8.5—9.8)	11.3 (10.8—11.8)	9.7 (8.8—10.7)	20.7 (17.8—23.5)	10.5 (9.5—11.5)	16.7 (15.0—18.4)	7.1 (6.1—8.1)	11.9 (10.4—13.3)
2017	9.9 (9.2—10.6)	11.4 (10.9—11.9)	9.9 (8.9—10.8)	22.3 (19.4—25.2)	11.3 (10.3—12.4)	17.0 (15.3—18.7)	7.7 (6.7—8.7)	11.3 (9.9—12.8)
2018	9.2 (8.5—9.8)	12.1 (11.6—12.6)	11.9 (10.8—12.9)	18.9 (16.2—21.6)	10.5 (9.6—11.5)	17.7 (16.0—19.5)	6.5 (5.5—7.4)	16.2 (14.5—17.9)
2019	10.9 (10.2—11.6)	12.6 (12.1—13.1)	10.6 (9.6—11.6)	20.9 (18.1—23.8)	12.1 (11.0—13.1)	18.1 (16.4—19.9)	7.0 (6.1—8.0)	13.2 (11.6—14.7)
2020	9.9 (9.2—10.6)	11.9 (11.4—12.4)	11.2 (10.2—12.3)	19.3 (16.5—22.0)	10.9 (9.9—11.9)	15.5 (13.9—17.2)	6.4 (5.5—7.3)	14.5 (12.9—16.1)
2021	10.7 (10.0—11.5)	11.3 (10.8—11.7)	11.6 (10.6—12.7)	17.5 (14.8—20.1)	10.6 (9.6—11.6)	16.8 (15.1—18.5)	5.8 (5.0—6.7)	11.9 (10.4—13.4)
2022	9.9 (9.2—10.5)	12.2 (11.7—12.7)	12.5 (11.4—13.5)	17.7 (15.1—20.3)	10.6 (9.6—11.6)	17.5 (15.8—19.3)	6.1 (5.2—7.0)	13.5 (11.9—15.0)

**Fig. 4:**
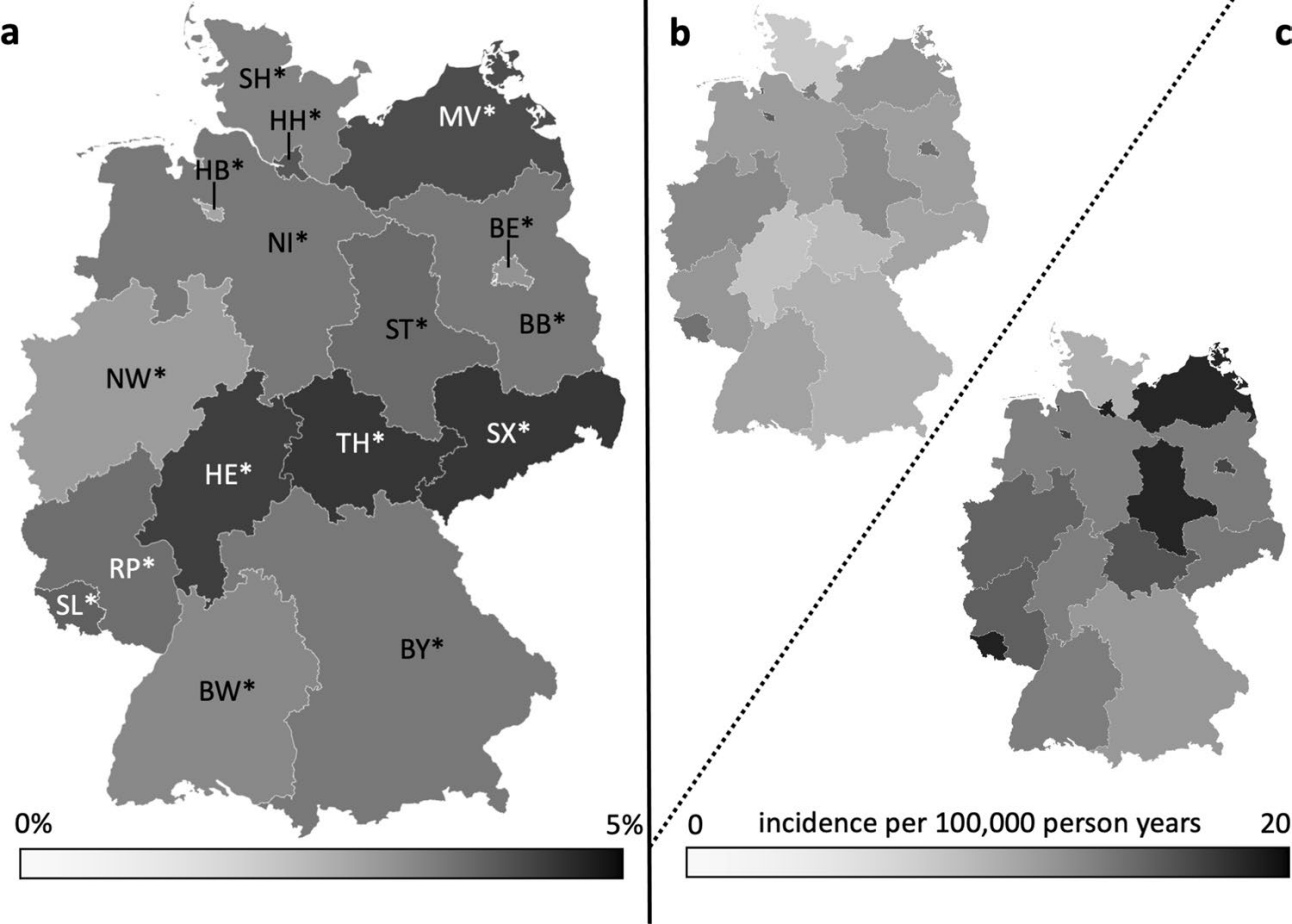
**a** Increase of the incidence of MSI-associated procedures. Annual percentage increase as result from the fully adjusted Poisson models stratified by federal state. Germany, 2005–2022. **b** Baseline incidence of MSI-associated procedures standardized for age and gender, Germany, 2005. **c** Latest Incidence of MSI-associated procedures standardized for age and gender, Germany, 2022. *Significant increase (*p*-value time trend Poisson-Regression model < 0.05); SH: Schleswig Holstein; MV: Mecklenburg-Vorpommern; HH: Hamburg; HB: Bremen; NI: Lower saxony; BE: Berlin; BB: Brandenburg; ST: Saxony-Anhalt; NW: North Rhine-Westphalia; HE: Hesse; TH: Thuringia; SX: Saxony; RP: Rhineland-Palatinate; SL: Saarland; BW: Baden-Wurttemberg; BY: Bavaria

The results of the time trend of incidence from the fully adjusted Poisson models are shown in Table 4, Table 5 and Fig. 4. We observed a marked increase in the incidence of MSI-associated procedures with a significant annual percentage increase of 2.8% in the observational period (relative risk per calendar year 1.028; 95% CI: 1.027–1.030, *p*-value < 0.001). The increase was slightly weaker in males (2.6% per year, RR: 1.026; 1.024–1.028; *p*-value < 0.001) than in females (3.2% per year, RR:1.032; 1.029–1.034; *p* < 0.001), although the baseline incidence was higher in males. Significant increases were observed in age groups 15–34 years (1.6% per year, RR: 1.016; 1.012 – 1.019; *p* < 0.001), 35–59 years (2.9% per year, RR: 1.029; 1.027 – 1.032; *p* < 0.001), 60–79 years (3.4% per year, RR: 1.039; 1.035 – 1.042; *p* < 0.001) and over 80 years (4.5% per year, RR: 1.045; 1.040 – 1.049; *p* < 0.001). In contrast no significant change was seen in children from 0 to 14 years (− 0.2% per year, RR: 0.998; 0.992 – 1.005; *p* = 0.63). A significant increase of MSI-associated procedures was found in all 16 federal states ranging from 1.7% per year in Bremen (RR: 1.017; 1.012–1.022; *p* < 0.001) to 4.0% per year in Saxony (RR: 1.040; 1.033–1.047; *p* < 0.001) and Thuringia (RR: 1.040; 1.034–1.046; *p* < 0.001).

**Table 4:** Results of the Poisson models for total population and stratified by gender and age group (Germany, 2005–2022)

	Total population	Gender		Age group				
		Male	Female	0–14	15–34	35–59	60–79	80 +
Risk factor								
Calendar year	1.028 (1.027—1.030)*	1.026 (1.024—1.028)*	1.032 (1.029—1.034)*	0.998 (0.992—1.005)	1.016 (1.012—1.019)*	1.029 (1.027—1.032)*	1.039 (1.035—1.042)*	1.045 (1.040—1.049)*
Gender ^a^	1.435 (1.413—1.458)*	-	-	1.226 (1.147—1.31)*	1.688 (1.631—1.748)*	1.684 (1.643—1.727)*	1.256 (1.213—1.301)*	0.883 (0.846—0.922)*
Age group								
0–14	0.503 (0.485—0.522)*	0.441 (0.42—0.464)*	0.608 (0.574—0.643)*	-	-	-	-	-
15–34	1.000^a^	1.000^a^	1.000^a^	-	-	-	-	-
35–59	0.957 (0.938—0.977)*	0.956 (0.932—0.982)*	0.959 (0.927—0.992)*	-	-	-	-	-
60–79	0.919 (0.898—0.942)*	0.815 (0.79—0.841)*	1.096 (1.055—1.138)*	-	-	-	-	-
80 +	1.194 (1.162—1.227)*	0.891 (0.858—0.926)*	1.704 (1.637—1.774)*	-	-	-	-	-
Federal state								
Baden-Württemberg	1.000^a^	1.000^a^	1.000^a^	1.000^a^	1.000^a^	1.000^a^	1.000^a^	1.000^a^
Bavaria	0.709 (0.674—0.746)*	0.706 (0.661—0.755)*	0.714 (0.66—0.772)*	0.645 (0.522—0.798)*	0.724 (0.642—0.818)*	0.71 (0.655—0.77)*	0.706 (0.635—0.785)*	0.721 (0.633—0.821)*
Berlin	1.232 (1.179—1.287)*	1.254 (1.184—1.328)*	1.2 (1.121—1.285)*	1.217 (1.017—1.456)*	1.458 (1.317—1.615)*	1.349 (1.26—1.446)*	1.066 (0.969—1.172)	0.922 (0.817—1.042)
Brandenburg	0.868 (0.827—0.911)*	0.892 (0.838—0.949)*	0.835 (0.775—0.9)*	0.866 (0.713—1.053)	1.279 (1.152—1.42)*	0.864 (0.8—0.933)*	0.65 (0.583—0.725)*	0.738 (0.648—0.839)*
Bremen	1.582 (1.518—1.650)*	1.556 (1.473—1.644)*	1.619 (1.519—1.726)*	1.217 (1.017—1.456)*	1.724 (1.562—1.903)*	1.741 (1.631—1.859)*	1.34 (1.224—1.466)*	1.521 (1.365—1.696)*
Hamburg	1.526 (1.463—1.591)*	1.506 (1.426—1.592)*	1.552 (1.455—1.656)*	1.516 (1.277—1.8)*	1.651 (1.494—1.823)*	1.545 (1.445—1.653)*	1.473 (1.349—1.61)*	1.414 (1.267—1.579)*
Hesse	0.824 (0.785—0.865)*	0.825 (0.775—0.88)*	0.823 (0.763—0.887)*	0.622 (0.502—0.771)*	0.933 (0.833—1.044)	0.872 (0.808—0.942)*	0.774 (0.698—0.859)*	0.732 (0.643—0.833)*
Mecklenburg-Vorpommern	1.439 (1.379—1.501)*	1.556 (1.473—1.644)*	1.276 (1.193—1.365)*	1.028 (0.852—1.239)	2.038 (1.852—2.243)*	1.545 (1.445—1.653)*	1.15 (1.048—1.263)*	1.072 (0.954—1.205)
Lower Saxony	0.904 (0.862—0.947)*	0.896 (0.842—0.954)*	0.914 (0.85—0.983)*	0.954 (0.789—1.154)	1.103 (0.989—1.229)	0.918 (0.851—0.99)*	0.791 (0.714—0.877)*	0.789 (0.695—0.896)*
North Rhine-Westphalia	1.103 (1.054—1.153)*	1.071 (1.009—1.136)*	1.147 (1.071—1.229)*	0.788 (0.645—0.963)*	1.298 (1.169—1.441)*	1.162 (1.082—1.248)*	1.002 (0.91—1.104)	1.002 (0.889—1.129)
Rhineland-Palatinate	1.002 (0.957—1.049)	1.001 (0.942—1.063)	1.004 (0.935—1.078)	0.783 (0.641—0.958)*	1.192 (1.072—1.326)*	1.051 (0.977—1.131)	0.898 (0.813—0.992)*	0.902 (0.798—1.019)
Saarland	1.637 (1.571—1.706)*	1.69 (1.601—1.783)*	1.565 (1.467—1.669)*	1.903 (1.615—2.243)*	2.167 (1.971—2.382)*	1.722 (1.612—1.838)*	1.34 (1.224—1.466)*	1.153 (1.028 – 1.294)*
Saxony	0.917 (0.874—0.961)*	0.945 (0.889—1.005)	0.877 (0.815—0.944)*	0.793 (0.649—0.968)*	1.147 (1.031—1.277)*	0.969 (0.899—1.044)	0.772 (0.696—0.856)*	0.786 (0.692—0.892)*
Saxony-Anhalt	1.470 (1.410—1.534)*	1.529 (1.447—1.616)*	1.389 (1.3—1.484)*	1.175 (0.981—1.408)	2.263 (2.059—2.486)*	1.591 (1.488—1.701)*	1.039 (0.944—1.143)	1.018 (0.905 – 1.147)
Schleswig–Holstein	0.576 (0.546—0.608)*	0.595 (0.554—0.638)*	0.55 (0.505—0.598)*	0.585 (0.47—0.729)*	0.769 (0.683—0.866)*	0.599 (0.55—0.653)*	0.483 (0.429—0.544)*	0.429 (0.368—0.500)*
Thuringia	1.136 (1.086—1.188)*	1.184 (1.117—1.255)*	1.069 (0.997—1.147)	0.949 (0.785—1.149)	1.423 (1.285—1.576)*	1.173 (1.093—1.26)*	0.951 (0.863—1.049)	1.063 (0.945—1.195)

Relative risk for severe MSI (95% Confidence interval). * *p*-value < 0.05;

^a^ Baseline: gender: female, age:15–34 years, federal state: Baden-Württemberg

**Table 5:** Results of the Poisson models stratified by federal state (Germany, 2005–2022). Relative risk for severe MSI (95% Confidence interval)

	Federal state							
	Baden-Württemberg	Bavaria	Berlin	Brandenburg	Bremen	Hamburg	Hesse	Mecklenburg-Vorpommern
Risk factor								
Calendar year	1.023 (1.016—1.029)*	1.026 (1.018—1.033)*	1.021 (1.015—1.026)*	1.026 (1.019—1.033)*	1.017 (1.012—1.022)*	1.033 (1.027—1.038)*	1.038 (1.030—1.045)*	1.035 (1.030—1.041)*
Gender ^a^	1.382 (1.294—1.477)*	1.367 (1.264—1.479)*	1.444 (1.360—1.533)*	1.476 (1.374—1.585)*	1.329 (1.261—1.400)*	1.341 (1.272—1.415)*	1.387 (1.290—1.492)*	1.685 (1.593—1.783)*
Age group								
0–14	0.696 (0.596—0.812)*	0.619 (0.512—0.749)*	0.58 (0.506—0.665)*	0.471 (0.402—0.552)*	0.491 (0.429—0.561)*	0.639 (0.564—0.723)*	0.464 (0.385—0.559)*	0.351 (0.304—0.404)*
15–34	1.000^a^	1.000^a^	1.000^a^	1.000^a^	1.000^a^	1.000^a^	1.000^a^	1.000^a^
35–59	1.128 (1.027—1.240)*	1.106 (0.990—1.236)	1.044 (0.965—1.130)	0.762 (0.697—0.833)*	1.139 (1.061—1.224)*	1.056 (0.981—1.138)	1.055 (0.956—1.164)	0.855 (0.798—0.917)*
60–79	1.321 (1.190—1.465)*	1.288 (1.139—1.456)*	0.965 (0.879—1.059)	0.672 (0.602—0.749)*	1.026 (0.943—1.116)	1.179 (1.085—1.281)*	1.096 (0.980—1.227)	0.745 (0.685—0.811)*
80 +	1.734 (1.545—1.946)*	1.726 (1.507—1.976)*	1.097 (0.983—1.223)	1.000 (0.887—1.128)	1.530 (1.397—1.675)*	1.485 (1.353—1.631)*	1.361 (1.198—1.546)*	0.912 (0.827—1.006)
	Federal state							
	Lower Saxony	North Rhine-Westphalia	Rhineland-Palatinate	Saarland	Saxony	Saxony-Anhalt	Schleswig–Holstein	Thuringia
Risk factor								
Calendar year	1.026 (1.020—1.033)*	1.019 (1.013—1.026)*	1.028 (1.021—1.034)*	1.031 (1.026—1.036)*	1.04 (1.033—1.047)*	1.029 (1.024—1.035)*	1.024 (1.016—1.033)*	1.040 (1.034—1.046)*
Gender ^a^	1.355 (1.264—1.452)*	1.290 (1.212—1.374)*	1.378 (1.290—1.472)*	1.492 (1.417—1.572)*	1.489 (1.389—1.596)*	1.522 (1.441—1.608)*	1.495 (1.37—1.632)*	1.531 (1.438—1.630)*
Age group								
0–14	0.602 (0.515—0.703)*	0.422 (0.358—0.498)*	0.457 (0.387—0.540)*	0.611 (0.547—0.682)*	0.48 (0.407—0.567)*	0.361 (0.316—0.413)*	0.529 (0.435—0.643)*	0.464 (0.399—0.54)*
15–34	1.000^a^	1.000^a^	1.000^a^	1.000^a^	1.000^a^	1.000^a^	1.000^a^	1.000^a^
35–59	0.939 (0.856—1.030)	1.010 (0.928—1.099)	0.995 (0.911—1.086)	0.896 (0.839—0.958)*	0.953 (0.87—1.043)	0.793 (0.742—0.848)*	0.879 (0.786—0.983)*	0.930 (0.857—1.009)
60–79	0.948 (0.851—1.055)	1.020 (0.926—1.124)	0.995 (0.898—1.101)	0.817 (0.754—0.884)*	0.888 (0.798—0.988)*	0.606 (0.557—0.66)*	0.829 (0.726—0.947)*	0.883 (0.802—0.972)*
80 +	1.241 (1.100—1.401)*	1.338 (1.200—1.492)*	1.312 (1.170—1.470)*	0.923 (0.840—1.015)	1.187 (1.053—1.339)*	0.780 (0.707—0.861)*	0.967 (0.826—1.131)	1.295 (1.166—1.438)*

* *p*-value < 0.05; ^a^ Baseline: gender: female, age:15–34 years

## Discussion

Despite good access to dental care, availability of antibiotic therapy and good socioeconomic conditions, severe MSI is a persistent challenge in general and oral healthcare. Its main cause is end stage dentoalveolar disease leading from caries, periodontal disease to cellulitis and abscess formation. It is still an important cause of hospitalization in oral and maxillofacial surgery departments [13]. However, reliable population-based data about long-term trends, age and gender distributions in the incidence of severe MSI are limited and may be inconclusive due to its different causes and clinical presentations. Most available data were collected from single center case series and therefore may not be representative. To our knowledge, this is the first population-based study to investigate the incidence of severe MSI using Germany’s DRG inpatient billing data base, which includes data on the entire population of Germany.

During the observational period from 2005–2022 we were able to detect a significant increase in the incidence of severe MSI requiring external incision and drainage in Germany. The phenomenon of rising incidence of severe MSI affects the entire society and is not limited to distinct regions, genders or age groups. These results were in accordance with other data describing an increase in maxillofacial infections. Robertson et al. report an increasing number of hospital admissions due to odontogenic infections in the hospitals of National Health System in the United Kingdom rising from 1.71 per 100,000 person years in the year 2000 to 5.36 per 100,000 person years in 2020 [15]. Similar data for the UK were published by Thomas et al. in 2008 [16]. Allareddy et al. analyzed 29,228 hospitalizations due to oral infections between 2004 and 2010 in the USA with an increasing number over time [17]. Seppänen et al. describe an increase of maxillofacial space infections requiring hospital care in the Helsinki and Uusimaa Hospital District in Finland representing more than 25% of the population in Finland with an increase from 5.3 per 100,000 inhabitants in 1995 to 7.2 per 100,000 inhabitants in 2005 [18]. Yang et al. saw a slight increase in head and neck space infections in Taiwan from 2.47 per 100,000 person years in 2007 to 3.71 per 100,000 person years 2016 [19].

Odontogenic infections are the main cause of MSI and can usually be found in more than 80% of all cases [20–22]. The caries experience among younger adults (35–44 years) in Germany has declined from 16.1% in 1997 and 14.6% in 2005 to 11.2% in the last national oral health study in 2014 [23–25]. Also caries experience in younger seniors (65–74 years) has declined from 23.6% in 1997 and 22.1% in 2005 to 17.7% in 2014 [23–25]. At first sight the improvement in societal oral health is contractionary to the increase in severe MSI. The increase might be more associated to individual social subgroups. Caries prevalence, number of missing teeth and edentulism as measures of oral health are significantly associated with lower income and lower education in Germany [26]. The association of these social factors with oral health and reduced utilization patterns increased over the last decades [27]. This may lead to a social disparity in oral health and, as a result, to an increase in severe infections in most disadvantaged groups. This is in alignment with data from the UK by Moles et al. reporting an increasing incidence of dental abscesses which is mostly driven by poorer people and strongly associated with the relative deprivation of the area of residence [28].

The phenomenon of rising incidence of severe MSI s not limited to individual genders or age groups. Nevertheless, some epidemiological factors are more predisposing than others. Male gender is an important predisposing factor as there is a consistent male preponderance in every study year which is in accordance with the literature showing that odontogenic infection are more prevalent in males [21, 29–31]. We also found incidence differences between age groups that were observed consistently over the 18-year observational period. Elderly people over 80 years of age have the highest incidence rates of severe MSI and they also share the strongest increase over time followed by older adults between 60 und 79 years. Seemingly contradictory at first glance, dental diseases including caries, tooth loss and edentulism decline in the German society as well as globally [32, 33]. This means a higher number of teeth is preserved in elderly people. There they are “teeth at risk” for dental disease and may act as a source of infection, especially when accompanied by systemic diseases associated with age such as dementia, tremor, diabetes, which have a negative impact on personal oral hygiene and oral health [34]. Together with a lower level of medical utilization due to limited mobility, this may result in a situation that is predisposing to advanced maxillofacial infections. This could be possibly a contributing factor for the increase in the number of MSI in these age groups. In patients with systemic diseases, the spread of the odontogenic infection requiring inpatient treatment and the occurrence of complications during the course of the disease are more common than in patients without systemic diseases [12, 18, 35, 36]. As systemic risk factors and comorbidities like diabetes, obesity, cardiovascular disease and mental disorders are on the rise, the risk for severe MSI may also increase over time especially in the age groups most affected by these conditions [37–40]. Further studies are needed to identify potential risk factors and risk groups for severe odontogenic infections [41].

MSI are usually caused by a polymicrobial variety of aerobic and anaerobic bacteria [20, 21, 42, 43]. A shift in the microbiological composition within odontogenic infections have been determined in preceding studies [42, 44]. Streptococcus species are the most frequently identified bacteria in MSI followed by Prevotella species and Staphylococcus species [45–47]. Antibiotic resistance is a common phenomenon in odontogenic infections with prevalence of more than 40% to at least one antibiotic agent [45, 48]. Accordingly, increased attention must be paid to the initial empiric antibiotic therapy. Penicillin-group antibiotics continue to be highly effective against Streptococci, Prevotella and Staphylococci while Clindamycin could not be shown to be effective as an empirical drug of choice for a high number of odontogenic infections [20, 29, 49]. In a recent study on antimicrobial resistances in oral- and maxillofacial infections in more than 20,000 clinical isolates from dental and oral-maxillofacial clinical settings in Germany Meinen et al. found high clindamycin and macrolide resistance proportions (> 17%) in Streptococcus spp. and Staphylococcus aureus isolates [48]. Moreover increasing resistance against clindamycin is an emerging problem in different bacteria and along different clinical settings [50–55]. The German S3-guideline on odontogenic infections recommends clindamycin only as a second-line antibiotic in the presence of an allergy to penicillin group antibiotics [56]. Nevertheless clindamycin is with more than 23.4% of all antibiotic prescriptions still the second most prescribed antibiotic among dentists in Germany [57]. In contrast to Germany clindamycin is rarely dentally prescribed in other industrialized countries like Great Britain, Norway or Canada [58–60]. Increased resistance rates for clindamycin require clarification among dentists in order to prescribe antibiotics according to national and international guidelines and the current scientific knowledge.

Increasing number of MSI requiring inpatient treatment with external incision leads to an increasing burden for the health system. There is only scarce data on costs caused by odontogenic infections in Germany [61]. Most Studies on this topic were conducted in the USA and Great Britain [62–64]. Due to the differences in the health systems, it is only insufficiently possible to transfer the results to Germany. Nevertheless, with an increasing incidence of inpatient treatments of MSI a relevant increase in financial expenditure for these conditions seems likely. Since reimbursement by statutory health insurance cannot fully cover the costs of treating these patients, this type of disease represents an economic burden not only for the cost bearer but also for the treating department [61].

This study is subject to certain strengths and limitations, which are mainly related to the dataset that was available for analysis. First, the specific reimbursed OPS codes from the national DRG system database only represent MSI treated by external incision and drainage in the analyzed years. MSI treated non-surgically are not included in the dataset. Second, the study only includes MSI cases requiring a hospital admission that led to the billing of a DRG. Patients treated as outpatients or who use other reimbursement schemes could not be included.

Since statutory health insurance in Germany provides a fully accessible and affordable healthcare system for every citizen and MSI is a serious and progressive disease that requires immediate medical attention and hospitalization, we assume that we can cover the vast majority of the German population and provide a comprehensive overview. Selection bias in MSI determination due to socioeconomic status is limited by the population-based approach. The completeness of our description is only limited by the dataset itself, which provides information at only an aggregated level and in limited detail without information about a specific cause or severity of the infection; therefore, a more advanced analysis including anamnestic and diagnostic information was not possible. This would, however, be interesting to evaluate the reasons for the increasing rate of severe MSI further. Nevertheless, the DRG inpatient billing data base provides an excellent tool for German researchers to perform large-scale clinical epidemiology investigations on procedures and diseases. The use of claims data also avoided recall bias and misclassification bias that may result from studies based on self-reported data.

To the best of our knowledge, as initially aimed, this study is the first to examine the incidence, time trends and epidemiological distribution of severe MSI at a national level in Germany after more than twenty years. It can contribute to an analysis on the influence of population structure and oral health care on the occurrence of severe MSI. The description of negative effects can contribute to the future addressing of risk groups who may take profit from early oral health care motivation or intervention. For this purpose, the investigation of the direct influence of social background should be expanded.

## Conclusion

The study suggests an increasing incidence rate of severe MSI in Germany over the last decades. This is not limited to certain regions, genders or age groups. Elderly patients over 80 years of age represent the social group that poses the greatest challenge. They present the highest increase in incidence over time and are beyond this the fastest growing age group in an aging society. Thus, severe MSI is an increasing challenge even in countries with comprehensive health care like Germany. As severe MSI is a promising target for prevention there have to be more focus on early detection and management of odontogenic infections.

## Data Availability

No datasets were generated or analysed during the current study.
